# Supplementary material for: DeepSNVMiner: a sequence analysis tool to detect emergent, rare mutations in subsets of cell populations
Source: PeerJ. 2016 May 24;4:e2074. doi: 10.7717/peerj.2074 (PMC4888318; doi:10.7717/peerj.2074)
Supplement: Supplemental Information 1 [file peerj-04-2074-s001.docx]

**Table S1**: Variant caller commands

| **Variant caller** | **Version** | **Command** |
| --- | --- | --- |
| DeepSNVMiner | 1.0 | run_deepsnv.pl -read1_fastq R1.fq -read2_fastq R2.fq -coord_bed chr22.bed -filename_stub test |
| FreeBayes | 1.0.2-6 | freebayes -f chr22.fa bam_file > freebayes.vcf |
| GATK | 3.2.2 | 1) java -jar GenomeAnalysisTK.jar -T HaplotypeCaller -R chr22.fa -L chr22.intervals -I bam_file -o gatk.gvcf -variant_index_type LINEAR -variant_index_parameter 128000 --minPruning 3 -ERC GVCF -contamination 0.0 --maxNumHaplotypesInPopulation 200 --max_alternate_alleles 3  2) java -jar -T GenotypeGVCFs -R chr22.fa -L chr.intervals -V gatk.gvcf-o gatk.vcf |
| LoFreq | 2.1.2 | lofreq call -f chr22.fa -o lofreq.vcf bam_file |
| SAMTools | 0.1.19 | samtools mpileup -C50 -uDEf chr22.fa bam_file \| bcftools view -vcg - > sam.vcf |

Variant caller commands utilized in our example. The commands listed match the exact commands run with the exception of the shortening of file names. The commands were chosen by either following documentation suggestions, or else by using default options.

**Table S2**: False positive rates for variant callers at increasing dilution levels

| **Dilution Percent** | **Total Variants** | **Deep-**  **SNVMiner** | **FreeBayes** | **GATK** | **LoFreq** | **SAMTools** |
| --- | --- | --- | --- | --- | --- | --- |
| 0 | 799962 | 0.014 | 0.16 | 34.05 | 0.18 | 31.94 |
| 50 | 408518 | 0.012 | 0.15 | 29.43 | 0.18 | 30.11 |
| 90 | 81708 | 0.012 | 0.17 | 28.22 | 0.21 | 26.09 |
| 99 | 8211 | 0.043 | 0.21 | 15.78 | 0.17 | 46.15 |
| 99.9 | 811 | 0.149 | 0.18 | 16.33 | 3.45 | 0 |
| 99.99 | 74 | 0 | 1.72 | 30.14 | 21.43 | N/A |
| 99.999 | 8 | 0 | 0 | 50.00 | 50.00 | N/A |
| 99.9999 | 2 | 0 | N/A | 0 | N/A | N/A |

False positive rates for DeepSNVMiner compared to FreeBayes, GATK, LoFreq, and SAMTools at increasing variant dilutions.

**Table S3**: False negative rates for variant callers at increasing dilution levels

| **Dilution Percent** | **Total Variants** | **Deep- SNVMiner** | **FreeBayes** | **GATK** | **LoFreq** | **SAMTools** |
| --- | --- | --- | --- | --- | --- | --- |
| 0 | 799962 | 41.74 | 31.35 | 71.35 | 66.52 | 99.85 |
| 50 | 408518 | 29.76 | 31.79 | 63.48 | 68.40 | 99.89 |
| 90 | 81708 | 17.83 | 31.45 | 33.86 | 77.41 | 99.90 |
| 99 | 8211 | 15.59 | 32.03 | 18.32 | 78.32 | 99.91 |
| 99.9 | 811 | 17.39 | 31.94 | 17.26 | 79.28 | 99.88 |
| 99.99 | 74 | 9.46 | 22.97 | 31.08 | 70.27 | 100 |
| 99.999 | 8 | 0 | 25.00 | 25.00 | 50.00 | 100 |
| 99.9999 | 2 | 0 | 100 | 0 | 0.00 | 100 |

False negative rates for DeepSNVMiner compared to FreeBayes, GATK, LoFreq, and SAMTools at increasing variant dilutions.

**Table S4**: Dilution series for cell lines HEK293 and OCI-LY10

| **Sample** | **HEK293 wt MYD88** | **OCI-LY10 L265P MYD88** |
| --- | --- | --- |
| Sample1 | 0% | 100% |
| Sample2 | 90% | 10% |
| Sample3 | 99% | 1% |
| Sample4 | 99.9% | 0.1% |
| Sample5 | 99.99% | 0.01% |
| Sample6 | 99.999% | 0.001% |
| Sample7 | 99.9999% | 0.0001% |
| Sample8 | 99.99999% | 0.00001% |
| Sample9 | 99.999999% | 0.000001% |
| Sample10 | 100% | 0% |

To measure the sensitivity of DeepSNVMiner a dilution series was performed with genomic DNA from two cells lines: (i) HEK293 (Human Embryonic Kidney): wild-type MYD88 (ii) OCI-LY10 (Ontario Cancer Institute, lymphoma cell line 10): heterozygous L265P MYD88 mutation.
